# Increased cardiovascular mortality in patients with mechanically expandable transcatheter aortic valve and without permanent pacemaker

**DOI:** 10.1136/openhrt-2023-002386

**Published:** 2023-12-13

**Authors:** Petr Hájek, Martin Horvath, Eva Hansvenclova, Monika Pecková, Radka Adlova

**Affiliations:** 1 Department of Cardiology, Second Faculty of Medicine, Charles University, Motol University Hospital, Prague, Czech Republic; 2 Institute of Applied Mathematics and Information Technologies, Faculty of Science, Charles University, Prague, Czech Republic

**Keywords:** Transcatheter Aortic Valve Replacement, Aortic Valve Stenosis, Death, Sudden, Cardiac, Pacemaker, Artificial

## Abstract

**Introduction:**

Use of the mechanically expandable transcatheter aortic valve (MEV) has been recently linked to increased risks of valve dysfunction and cardiovascular mortality. The risk of developing conduction disturbance with the MEV valve is well known, and the negative prognostic impact of permanent pacemaker implantation (PPI) after transcatheter aortic valve implantation is another consideration.

**Aim:**

This study aimed to compare the mid-term survival of patients with MEV and self-expandable valves (SEV), and to examine survival of both groups according to the presence or absence of PPI.

**Methods:**

This single-centre, retrospective, observational study examined data from MEV and SEV groups comprising 92 and 373 patients, respectively. The mean clinical follow-up was 2.5±1.7 years. Mortality information was obtained from the National Institutes of Health Information and Statistics.

**Results:**

Baseline characteristics were comparable between the groups. The log-rank test showed higher cardiovascular mortality in the MEV group (p=0.042; the relative risk (RR) 1.594 (95% CI 1.013 to 2.508)). The Cox proportional hazards model identified MEV implantation as an independent predictor of cardiovascular mortality. The rate of PPI was twice as high in the MEV vs SEV group (33.7% vs 16.1%; p<0.001). We compared the survival of both groups according to the presence or absence of PPI and found higher mortality in the MEV group without PPI versus the SEV group without PPI (p=0.007; RR 2.156 (95% CI 1.213 to 3.831)). Survival did not differ in the groups with PPI.

**Conclusions:**

A higher mid-term cardiovascular mortality rate was observed with MEV versus SEV implants. Comparing both groups according to the presence or absence of PPI, we observed a higher mortality risk in patients with MEV without PPI than in SEV without PPI. In contrast, mortality did not differ between the groups when PPI was implanted.

**WHAT IS ALREADY KNOWN ON THIS TOPIC**

- Use of the mechanically expandable transcatheter aortic valve (MEV) has been recently linked to increased risks of valve dysfunction and cardiovascular mortality. The risk of developing conduction disturbance with the MEV valve is well known, and the negative prognostic impact of permanent pacemaker implantation (PPI) after transcatheter aortic valve implantation is another consideration.

**WHAT THIS STUDY ADDS**

- Our study suggested that higher cardiovascular mortality was independently associated with the use of MEV versus self-expandable valves (SEV) implants. Comparing both groups according to the presence or absence of PPI, we observed a higher mortality risk in patients with MEV without PPI than in those with SEV without PPI.

**HOW THIS STUDY MIGHT AFFECT RESEARCH, PRACTICE OR POLICY**

- Although MEVs were recalled in 2020, thousands of patients have been treated with them. Therefore, patients with MEV without PPI deserve increased attention during long-term follow-up.

## Introduction

Recently published data have revealed a higher risk of bioprosthetic valve dysfunction of mechanically expandable valves (MEV) designed for transcatheter aortic valve implantation (TAVI).^1 2^ In fact, the results of our pilot study^3^ have suggested that higher cardiovascular mortality was independently associated with the use of MEV versus self-expandable transcatheter valves (SEV) over mid-term follow-up. Although MEVs were recalled in 2020^4 5^ and are not currently available for clinical use, more than 10 000 patients have been treated with them.^6^ To date, long-term MEV data have been published from only one randomised clinical trial.^6^ The authors confirmed a higher risk of prosthetic thrombosis with MEV implants, but they found comparable survival outcomes to those with SEV.

New permanent pacemaker implantation (PPI) has been studied as a potential risk factor for long-term survival after TAVI.^7^ Also, MEV implantation has been associated with a high risk of conduction disturbances.^8 9^ Because patients with MEV have constituted only a small part of the published meta-analyses,^7^ it is unclear whether the results regarding the effect of PPI on survival after TAVI are applicable.

### Aim

The aim of our study was to compare the mid-term survival of patients with MEV and SEV and to evaluate survival of both groups according to the presence or absence of PPI.

## Methods

### Design

We conducted a single-centre, retrospective, observational study comparing the outcomes of patients with severe aortic stenosis (AS) who underwent TAVI with mechanically expandable intra-annular Lotus and Lotus Edge (Boston Scientific, USA) versus self-expandable supra-annular Evolut R (Medtronic, USA) and Acurate Neo (Boston Scientific, USA) transcatheter valves.

### Patients and procedures

A total of 481 consecutive patients with severe AS who underwent TAVI between August 2015 and June 2022 were enrolled. On admission, patients were either haemodynamically stable for planned diagnostic evaluation or acutely decompensated with heart failure. Severe AS was diagnosed according to echocardiographic criteria. The indication for TAVI was established by consensus among members of the Heart Team. All TAVI procedures were performed by highly experienced interventional cardiologists at a single tertiary centre. Clinical, demographic and echocardiographic data were recorded at baseline and over follow-up. Serum troponin I levels were routinely analysed 24 hours after TAVI. The patients underwent post-TAVI clinical and echocardiographic examinations prior to hospital discharge, at 30 days, and at 1 year. Information regarding specific events was obtained from follow-up visits and the National Institutes of Health Information and Statistics.

The Lotus and Lotus Edge implants are bioprosthetic aortic valves comprising a braided nitinol wire frame with three bovine pericardial leaflets and a polymer membrane that surrounds the lower valve half to reduce paravalvular leaks. The valve is premounted on a delivery catheter and deployed via controlled mechanical expansion, enabling repositioning or retrieval of the valve at any point before its release.^10^ The novel features of the Lotus Edge system include increased flexibility of the delivery catheter, enhanced visualisation of the locking mechanism and Depth Guard technology designed to reduce left ventricular outflow tract (LVOT) interactions and potentially reduce PPI.^11^

The Medtronic CoreValve Evolut R System comprises the Evolut R valve and the EnVeo R Delivery Catheter System (DCS) with the InLine sheath. The trileaflet valve and sealing skirt are made of porcine pericardial tissue and sutured in a supra-annular position on a compressible and self-expandable nitinol frame. EnVeo R DCS enables the valve to be fully repositionable and recapturable before full release by turning the delivery handle.^12^ The Acurate Neo (Boston Scientific, USA) transcatheter valve consists of three porcine pericardial leaflets mounted on a self-expanding nitinol frame with an upper crown that provides supra-annular anchoring and caps the native leaflets, a waist that conforms to the native annulus, and a lower crown protruding few millimetres into the LVOT; an inner and outer porcine pericardium fabric skirt covers the inflow tract of the nitinol stent.^13^ All TAVI procedures were performed using the Lotus Introducer set (Boston Scientific).

### Outcomes

The primary outcomes were the mid-term all-cause and cardiovascular mortality rates. Secondary outcomes were clinical outcomes including periprocedural myocardial infarction; overt central nervous system injury at 30 days; bleeding complications types 2, 3 or 4 at 30 days; major vascular complications at 30 days; acute kidney failure stage 2 or 3; and valve malposition. Outcomes were defined according to standardised endpoint definitions for TAVI clinical trials in a consensus report from the Valve Academic Research Consortium.^14^

### Statistical analysis

Data are presented as mean±SD for continuous variables and as median±IQR for continuous variables with outliers. Continuous variables were compared using Welch’s two-sample t-tests for normally distributed variables and two-sample Wilcoxon tests for variables with some outliers. Categorical variables were compared using the Pearson χ^2^ test of independence or Fisher’s exact test (in case of small expected counts). A p<0.05 was considered statistically significant.

The log-rank test was used to compare survival times between the two groups. Kaplan-Meier estimates of the survival curves are presented. The Cox proportional hazards model was used to identify predictors of all-cause and cardiovascular mortality and to adjust for potential baseline differences between the groups. We analysed data including sex, age, presence of diabetes mellitus, arterial hypertension, atrial fibrillation, coronary artery disease, creatinine level before and after TAVI, haemoglobin and platelet levels before and after TAVI, Society of Thoracic Surgeons (STS) score, aortic valve gradient (AVG) before and after TAVI, ejection fraction, aortic valve area before TAVI, wall thickness, New York Heart Association classification before TAVI, necessity of acute TAVI, aortic regurgitation before TAVI, balloon valvuloplasty, amount of contrast dye during TAVI, open surgical access, pacemaker before and after TAVI, and type of TAVI valve. All analyses were performed using the statistical program R and GraphPad Prism V.6.05.

## Results

### Baseline characteristics

A total of 481 consecutive single-centre patients with symptomatic AS received the Lotus, Lotus Edge, Evolut R or Acurate Neo valves. Valve selection was made at the discretion of clinicians. Sixteen patients were excluded (3.3%; 12 patients with valve-in-valve procedure, four with pure aortic regurgitation). Therefore, our sample comprised 465 patients (72 Lotus and 20 Lotus Edge valves in the MEV group; and 167 Evolut R and 206 Acurate Neo valves in the SEV group). The baseline clinical and echocardiographic characteristics did not differ between groups, except for a slightly higher degree of left ventricle hypertrophy present in the MEV group (table 1).

**Table 1:** Clinical and echocardiographic characteristics

	SEV group All N=373	MEV group All N=92	P value	SEV group PM+ N=103	MEV group PM+ N=40	P value	SEV group PM− N=270	MEV group PM− N=52	P value
Clinical characteristics									
Age, years*	77.5±7.3	78.8±6.4	0.113	79.4±6.4	79.0±7.0	0.767	76.8±7.5	78.6±6.0	0.072
Males, n (%)	199 (53.4)	48 (52.2)	0.839	68 (66)	28 (70)	0.696	131 (48.5)	20 (38.5)	0.183
Body mass index*	29.0±5.3	28.7±4.7	0.610	28.4±5.1	28.9±5.0	0.541	29.2±5.4	28.5±4.5	0.314
NYHA class†	2.5±1.0	3.0±1.0	0.538	2.5±1.0	3.0±1.0	0.934	2.5±1.0	3.0±1.0	0.532
NYHA class ≥III, n (%)	165 (45.2)‡	50 (54.9)‡	0.096	47 (47.0)‡	21 (53.8)‡	0.468	118 (44.5)‡	29 (55.8)	0.137
Diabetes mellitus, n (%)	156 (41.9)	30 (32.6)	0.102	50 (48.5)	12 (30.0)	0.045	106 (39.4)	18 (34.6)	0.516
Arterial hypertension, n (%)	301 (80.7)	68 (73.9)	0.136	90 (87.4)	30 (75.0)	0.071	211 (78.4)	38 (73.1)	0.396
Atrial fibrillation, n (%)	126 (33.8)	35 (38.0)	0.441	55 (53.4)	21 (52.5)	0.923	71 (26.3)	14 (26.9)	0.925
Coronary artery disease, n (%)	172 (46.1)	37 (40.2)	0.308	49 (47.6)	20 (50.0)	0.794	123 (45.6)	17 (32.7)	0.087
Creatinine, µmol/l†	88±37	87±42	0.951	95±51	89±61	0.332	84±31	87±37	0.995
Haemoglobin, g/L*	125.5±17.6	125.9±16.1	0.814	125.4±17.8	126.7±17.4	0.704	125.5±17.6	125.4±15.2	0.950
Platelets, ×10^9^/L*	207±67	192±66	0.067	191±71	180±60	0.404	213±64	202±69	0.269
Pacemaker before TAVI, n (%)	43 (11.5)	9 (9.8)	0.715	43 (41.7)	9 (22.5)	0.032	–	–	–
LBBB before TAVI, n (%)	13 (3.5)	7 (7.6)	0.081	0	1 (2.5)	0.107	13 (4.8)	6 (11.5)	0.060
STS score†	2.4±2.7	2.2±2.3	0.350	2.7±3.3	2.3±2.4	0.140	2.2±2.5	2.1±2.3	0.583
Acute procedure, n (%)	38 (10.2)	7 (7.6)	0.454	9 (8.7)	2 (5.0)	0.452	29 (10.7)	5 (9.6)	0.809
Agatson score†	2441±1796	3213±2431	0.101	2637±1963	3316±2014	0.327	2382±1772	3088±2947	0.264
Echocardiography characteristics									
Mean gradient, mm Hg*	42±14	43±15	0.298	40±14	42±14	0.679	42±14	45±15	0.235
Aortic valve area, cm^2^*	0.8±0.2	0.7±0.2	0.175	0.8±0.2	0.8±0.2	0.480	0.8±0.2	0.7±0.2	0.166
Aortic regurgitation grade ≥3, n (%)	16 (4.4)‡	5 (5.4)	0.587	3 (3.0)‡	1 (2.5)	1.000	13 (4.9)‡	4 (7.7)	0.495
LVEF, %†	60±15	60±20	0.365	55±25	55±15	0.500	60±15	60±15	0.258
Septal wall thickness, mm*	12.6±2.1	13.2±2.0	0.014	12.8±2.1	13.3±2.2	0.195	12.5±2.1	13.1±1.9	0.048
Posterior wall thickness, mm*	11.8±1.6	12.4±1.5	<0.001	11.9±1.6	12.4±1.5	0.096	11.8±1.6	12.5±1.4	0.002
Mitral regurgitation grade ≥3, n (%)	39 (10.6)‡	7 (7.6)	0.397	19 (18.6)‡	4 (10.0)	0.209	20 (7.5)‡	3 (5.8)	0.660

*Plus-minus values are mean±SD.

†Plus-minus values are median±IQR.

‡Percentage is calculated from the available number of examinations performed.

LBBB, left bundle branch block; LVEF, left ventricular ejection fraction; MEV, mechanically expandable valve; NYHA, New York Heart Association; PM–, PM not implanted; PM+, permanent pacemaker implanted; SEV, self-expandable valve; STS, Society of Thoracic Surgeons score; TAVI, transcatheter aortic valve implantation.

### Procedural and postprocedural characteristics

In the SEV group, we used balloon valvuloplasty more often and a larger amount of contrast dye. Open surgical access was used more often in the MEV group. We observed a slight but significantly greater drop in haemoglobin and platelet counts in the MEV group. The mean AVG (AVGm) at 30 days was significantly higher in the MEV group, and this difference remained significant at 1 year (table 2).

**Table 2:** Procedural and postprocedural characteristics

	SEV group All N=373	MEV group All N=92	P value	SEV group PM+ N=103	MEV group PM+ N=40	P value	SEV group PM− N=270	MEV group PM− N=52	P value
Procedural characteristics									
Balloon valvuloplasty, n (%)	180 (48.3)	10 (11.1)	<0.001	47 (45.6)	5 (12.5)	<0.001	133 (49.3)	5 (10.0)	<0.001
Open surgical access, n (%)	118 (31.6)	72 (78.3)	<0.001	42 (40.8)	28 (70.0)	0.002	76 (28.1)	44 (84.6)	<0.001
Contrast dye, mL*	130±60	100±55	<0.001	135±70	110±77	0.195	130±60	100±50	<0.001
Postprocedural characteristics									
Troponin in 24 hours, ng/L*	333±593	634±769	<0.001	430±1034	648±532	0.076	266±542	605±1423	<0.001
Creatinine, µmol/L*	77±39	83±41	0.257	92±55	88±48	0.732	75±34	79±37	0.320
Haemoglobin, g/L†	115±18	111±19	0.056	114±18	111±17	0.236	115±18	111±21	0.161
Platelets, ×10^9^/L†	143±52	110±51	<0.001	137±54	102±47	<0.001	146±52	116±53	<0.001
AVB requiring PM implantation, n (%)									
Among all patients	60 (16.1)	31 (33.7)	<0.001	60 (58.3)	31 (77.5)	0.035	0	0	1.000
Among all patients without PM at baseline	60 (18.2)	31 (50.8)	<0.001	60 (100)	31 (100)	1.000	0	0	1.000
New onset of LBBB after TAVI, n (%)									
Among all patients	60 (16.1)	28 (30.4)	0.002	1 (1.0)	0	0.532	59 (21.9)	28 (53.8)	<0.001
Among patients without LBBB at baseline	60 (16.6)	28 (32.9)	<0.001	1 (1.0)	0	0.537	59 (23.0)	28 (60.9)	<0.001
Length of hospital stay after TAVI, days*	5±2	6±3	<0.001	6±4	7±4	0.016	5±2	6±2	<0.001
Echocardiographic characteristics at 30 days									
Mean gradient, mm Hg†	8±4	14±7	<0.001	8±3	13±6	<0.001	8±5	14±8	<0.001
Mean gradient ≥20, n (%)	2 (0.6)‡	11 (13.1)‡	<0.001	0‡	3 (8.6)‡	0.002	2 (0.8)‡	8 (16.3)‡	<0.001
Aortic regurgitation grade ≥3, n (%)	2 (0.6)‡	0‡	1.000	1 (1.1)‡	0‡	1.000	1 (0.4)‡	0‡	1.000
Echocardiographic characteristics at 1 year									
Mean gradient, mm Hg†	9±3	12±4	<0.001	8±3	11±4	0.002	9±3	12±4	<0.001
Mean gradient ≥20, n (%)	1 (0.5)‡	3 (4.9)‡	0.040	0‡	1 (3.8)‡	0.321	1 (0.7)‡	2 (5.7)‡	0.094
Aortic regurgitation grade ≥3, n (%)	1 (0.5)‡	0‡	1.000	0‡	0‡	1.000	1 (0.7)‡	0‡	1.000

*Plus-minus values are median±IQR.

†Plus-minus values are mean±SD.

‡Percentage is calculated from the available number of examinations performed.

AVB, atrioventricular block; LBBB, left bundle branch block; MEV, mechanically expandable valve; PM–, PM not implanted; PM+, permanent pacemaker implanted; SEV, self-expandable valve; TAVI, transcatheter aortic valve implantation.

In the MEV group, there were twofold rates of new PPI, new onset of left bundle branch block (NO-LBBB), and increased cardiac troponin I levels compared with the SEV group. In the SEV group, higher troponin I levels were observed in those with new PPI versus those without (430±1034 ng/L vs 266±542 ng/L, respectively; p=0.001). In the MEV group, this difference was not statistically significant (648±532 ng/L vs 605±1423 ng/L, respectively; p=0.916).

### Secondary outcomes

The rate of procedural complications was low and did not differ between the groups (table 3). In addition, we observed the same technical success in the MEV and SEV groups (96.7% vs 97.6%, respectively; p=0.712). The device success was numerically higher in the SEV group (92.2% SEV vs 85.9% MEV; p=0.057). This difference was the result of the higher incidence of AVGm >20 mm Hg in the MEV group (table 2). Significantly higher early safety composite endpoints were observed in the SEV group (77.4% SEV vs 57.6% MEV; p<0.001). This difference was mainly driven by higher rates of PPI in the MEV group (33.7% MEV vs 16.1% SEV; p<0.001) (table 2). All-cause and cardiovascular mortality at 30 days and over the first year did not significantly differ (table 3).

**Table 3:** Primary and secondary outcomes

	SEV group All N=373	MEV group All N=92	P value	SEV group PM+ N=103	MEV group PM+ N=40	P value	SEV group PM– N=270	MEV group PM– N=52	P value
Intraprocedural death, n (%)	1 (0.3)	0	1.000	0	0	1.000	1 (0.4)	0	1.000
Periprocedural myocardial infarction, n (%)	3 (1.0)*	2 (2.3)*	0.310	1 (1.1)*	0*	1.000	2 (0.9)*	2 (4.0)*	0.167
Myocardial injury not meeting MI criteria, n (%)	178 (59.3)*	70 (81.4)*	<0.001	62 (69.7)*	30 (83.3)*	0.178	116 (55.0)*	40 (80.0)*	0.001
Overt CNS injury at 30 days, n (%)	6 (1.6)	2 (2.2)	0.660	1 (1.0)	1 (2.5)	0.483	5 (1.9)	1 (1.9)	1.000
Bleeding complications type 2–4 at 30 days, n (%)	13 (3.5)	7 (7.6)	0.081	3 (2.9)	4 (10.0)	0.096	10 (3.7)	3 (5.8)	0.448
Acute kidney injury—stage 2 or 3, n (%)	1 (0.3)*	0*	1.000	1 (1.0)*	0	1.000	0*	0*	1.000
Major vascular complications on TAVI day, n (%)	3 (0.8)	2 (2.2)	0.258	2 (1.9)	0	1.000	1 (0.4)	2 (3.8)	0.069
Major vascular complications at 30 days, n (%)	1 (0.3)	2 (2.2)	0.101	1 (1.0)	0	1.000	0	2 (3.8)	0.026
Major non-vascular complications. on TAVI day, n (%)	0	0	1.000	0	0	1.000	0	0	1.000
Major non-vascular complications. at 30 days, n (%)	0	0	1.000	0	0	1.000	0	0	1.000
Major cardiac complications. on TAVI day, n (%)	0	1 (1.1)	0.198	0	1 (2.5)	0.280	0	0	1.000
Major cardiac complications. at 30 days, n (%)	4 (1.1)	0	1.000	1 (1.0)	0	1.000	3 (1.1)	0	1.000
Incorrect positioning of a single prosthesis, n (%)	6 (1.6)	0	0.604	3 (2.9)	0	0.096	3 (1.1)	0	1.000
Intervention related to the device or to a major vascular or access-related or cardiac structural complication, n (%)	3 (0.8)	1 (1.1)	0.587	1 (1.0)	1 (2.5)	0.483	2 (0.7)	0	1.000
All-cause mortality at 30 days, n (%)	12 (3.2)	3 (3.3)	1.000	3 (2.9)	1 (2.5)	1.000	9 (3.3)	2 (3.8)	0.694
All-cause mortality at 1 year, n (%)	50 (13.4)	14 (15.2)	0.773	20 (19.4)	6 (15.0)	0.452	30 (11.1)	8 (15.4)	0.445
Cardiovascular mortality at 1 year, n (%)	31 (8.3)	11 (12.0)	0.342	11 (10.7)	4 (10.0)	0.775	20 (7.4)	7 (13.5)	0.189
All-cause mortality during follow-up, n (%)	98 (26.3)	46 (50.0)	0.112	35 (34.0)	19 (47.5)	0.934	63 (23.3)	27 (51.9)	0.073
Cardiovascular mortality during follow-up, n (%)	58 (15.5)	31 (33.7)	0.042	22 (21.4)	12 (30.0)	0.902	36 (13.3)	19 (36.5)	0.007
Technical success, n (%)	364 (97.6)	89 (96.7)	0.712	99 (96.1)	39 (97.5)	1.000	265 (98.1)	50 (96.2)	0.315
Device success, n (%)	344 (92.2)	79 (85.9)	0.057	95 (92.2)	36 (90.0)	0.666	249 (92.2)	43 (82.7)	0.030
Early safety, n (%)	288 (77.4)	53 (57.6)	<0.001	39 (37.9)	7 (17.5)	0.019	249 (92.2)	46 (88.5)	0.370

*The calculation of the percentage is made from the available number of examinations performed.

CNS, central nervous system; MEV, mechanically expandable valve; PM+, permanent pacemaker implanted; PM–, PM not implanted; SEV, self-expandable valve; TAVI, transcatheter aortic valve implantation.

### Primary outcomes

Overall, 144 all-cause deaths occurred over 1175 patient-years (mean follow-up±SD = 2.5±1.7 years), which translates to 14.8 and 11.3 deaths per 100 patient-years in the MEV and SEV groups, respectively. The log-rank test showed higher mortality in the MEV group for all-cause deaths, but this difference did not reach statistical significance (p*=*0.112; the relative risk (RR) 1.345 (95% CI 0.932 to 1.940)).

**Figure 1:**
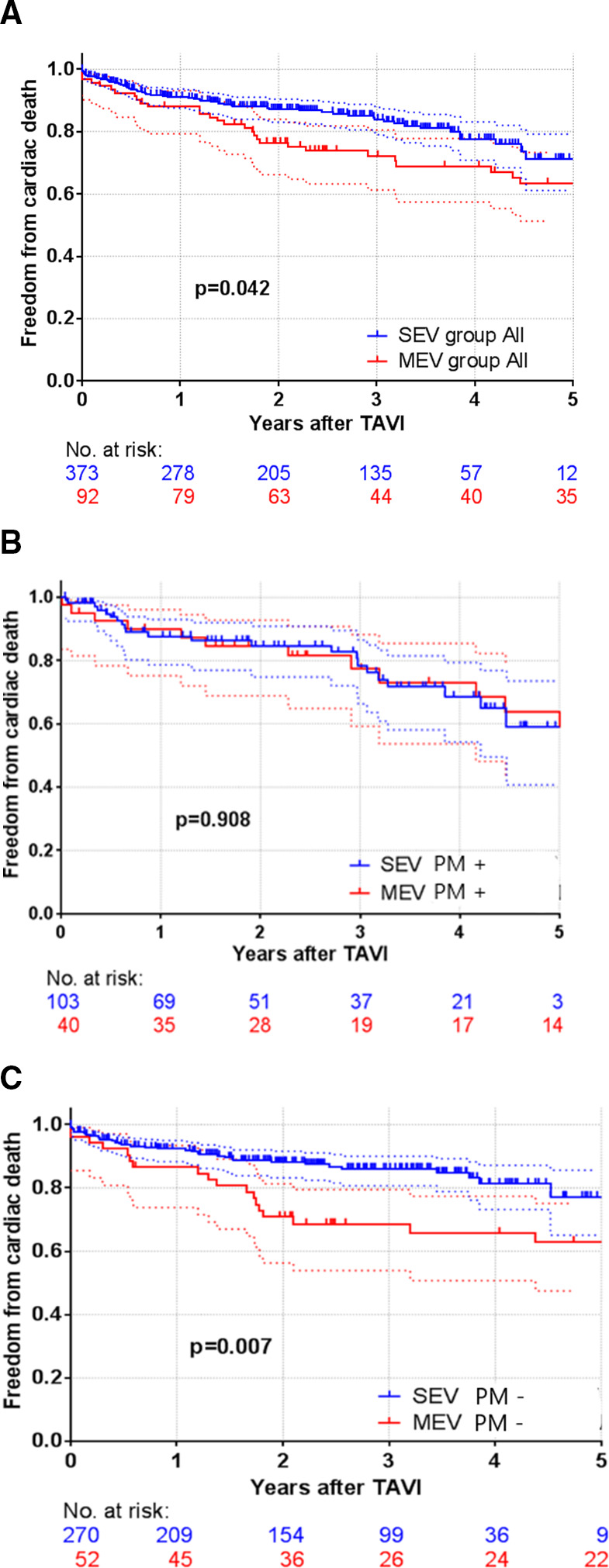
(A) Cardiovascular mortality for SEV and MEV. (B) Cardiovascular mortality for SEV and MEV with permanent pacemaker implanted. (C) Cardiovascular mortality for SEV and MEV without permanent pacemaker implanted. MEV, mechanically expandable valve; PM+, permanent pacemaker implanted; PM−, PM not implanted; SEV, self-expandable valve; TAVI, transcatheter aortic valve implantation.

A total of 89 cardiovascular deaths occurred over follow-up, which translates to 10.0 MEV and 6.7 SEV deaths per 100 patient-years. The log-rank test showed significantly higher cardiovascular mortality in the MEV group (p=0.042; RR 1.594 (95% CI 1.013 to 2.508)) (figure 1A).

### Subanalysis of the studied patients

Overall, the Cox proportional hazards model identified the following factors as predictors of cardiovascular mortality: MEV implantation (p=0.021; RR 1.71 (95% CI 1.08 to 2.71)), male sex (p=0.003; RR 1.93 (95% CI 1.25 to 2.96)), higher STS score (p<0.001; RR 1.05 (95% CI 1.02 to 1.07)) and lower haemoglobin level before TAVI (p<0.001; RR 0.98 (95% CI 0.97 to 0.99)). Patients of the same gender, with the same STS score and haemoglobin level before TAVI had a 1.7-fold higher risk of cardiovascular death with MEV than with SEV implants.

### Association of cardiovascular survival and pacemaker implantation after TAVI

We analysed the survival of all patients according to the presence or absence of PPI, regardless of the implanted valve type. A total of 89 cardiovascular deaths occurred over follow-up, which translates to 9.4 and 6.8 deaths per 100 patient-years in the groups with and without PPI, respectively. The log-rank test did not reveal any mortality difference (p=0.140; RR 1.38 (95% CI 0.9 to 2.2)).

We performed further survival analysis comparing the MEV and SEV groups regarding the presence or absence of PPI. A total of 34 cardiovascular deaths occurred over follow-up in patients with any PPI (PPI before TAVI and new PPI after TAVI), which translates to 9.1 and 9.6 deaths per 100 patient-years in the MEV and SEV groups, respectively. A total of 24 cardiovascular deaths occurred over follow-up in patients with new PPI after TAVI, which translates to 9 and 10.3 deaths per 100 patient-years in the MEV and SEV groups, respectively. The log-rank test did not show any difference in cardiovascular mortality between groups (any PPI: p=0.908; RR 0.961 (95% CI 0.476 to 1.928); new PPI after TAVI only: p=0.682; RR 0.847 (95% CI 0.372 to 1.889)) (figure 1B).

When we compared patients without PPI in the MEV and SEV groups, we found 55 cardiovascular deaths, which translates to 10.6 and 5.7 deaths per 100 patient-years in the MEV and SEV groups, respectively. The log-rank test showed significantly higher cardiovascular mortality in the MEV group (p=0.007; RR 2.156 (95% CI 1.213 to 3.831)) (figure 1C).

### Subanalysis of patients without PPI

In patients without PPI, the Cox proportional hazards model identified the following predictors of cardiovascular mortality: MEV valve implantation (p=0.002; RR 2.53 (95% CI 1.41 to 4.53)), history of coronary artery disease (p=0.041; RR 1.8 (95% CI 1.02 to 3.15)), elevated STS score (p=0.012; RR 1.04 (95% CI 1.01 to 1.06)) and lower haemoglobin level before TAVI (p=0.002; RR 0.97 (95% CI 0.96 to 0.99)). Patients with the same history of coronary artery disease, STS score and haemoglobin level before TAVI had a 2.5-fold higher risk of cardiovascular death with MEV than SEV implants.

## Discussion

Our single-centre, retrospective, observational study reported two essential findings: (1) the use of MEV was independently associated with higher cardiovascular mortality than that of SEV and (2) MEV patients without PPI had a higher mortality risk than SEV patients without PPI. However, no mortality difference between the two groups was observed when a permanent pacemaker was implanted.

The mechanically expandable TAVI valve remains the only fully retrievable prosthesis with excellent results regarding minimal paravalvular leaking.^10 15 16^ Although the valve is no longer commercially available, thousands of prostheses have been implanted.^6^ Therefore, evaluation of the long-term outcomes of this valve is of utmost importance. Last year, the results of one observational study with a mean follow-up of 36 months^2^ and those from a research correspondence with a median follow-up of 3.3 years^1^ have been published. Both studies are consistent with the evidence for higher prevalence of valve endocarditis and thrombosis compared with other transcatheter valves. It is unclear whether these unfavourable outcomes also result in increased mortality over long-term follow-up.

Recently, a secondary analysis of the REPRISE III randomised clinical trial was presented. In contrast with our results, the findings of that analysis suggest that, at the 5-year follow-up, the Lotus (ie, MEV) valve had comparable outcomes to those of the CoreValve/Evolut R (ie, SEV).^6^ In REPRISE III, 51.5% of SEV were prostheses of the first generation CoreValve, without the option to recapture and reposition, and in that regard unlike the second generation Evolut R. In the study by Giannini *et al*,^17^ when compared with patients receiving the CoreValve, those treated with the Evolut R valve showed a significant survival benefit at 1 year (HR 1.80, 95% CI 1.01 to 3.23, p=0.046) and a significantly lower risk of PPI (22.3% and 35.0% for Evolut R and CoreValve, respectively; p=0.008). We believe that a significant proportion of the first generation prostheses in REPRISE III could have adversely affected the survival rate of the SEV group and thus, have mitigated the mortality difference we observed in our study.

Patients with NO-LBBB or requiring PPI after TAVI have a higher risk of death and rehospitalisation for heart failure at 1 year^18^ and over long-term follow-up than those without NO-LBBB or not receiving PPI.^7 18^ This has been primarily studied in balloon-expandable valves or SEV, where no significant survival difference according to the valve type was observed. Few studies included MEV in their analyses.^7^ In the largest randomised study (REPRISE III)^19^ and the largest observational study^20^ comparing MEV and SEV, the need for postprocedural PPI did not significantly impact survival within 1 year. In our study, we did not observe the negative prognostic association of MEV with PPI (p=0.648; RR 0.832 (95% CI 0.387 to 1.804)) or NO-LBBB (p=0.259; RR 0.628 (95% CI 0.262 to 1.430)). This suggests that the association of survival with post-MEV PPI may vary from that with PPI after other types of bioprosthesis.

The increased risk of all-cause death among patients receiving PPI can be related to cardiac and non-cardiac causes.^7^ Regarding these, mechanical injury to the LVOT that occurs during TAVI can play a role.^21^ In our study, the Lotus valve caused an important mechanical injury to LVOT; this was documented by significantly higher number of new PPI (33.7% vs 16.1% in the MEV and SEV groups, respectively; p<0.001) and NO-LBBB (30.4% vs 16.1%, respectively; p*=*0.002). The long-lasting mechanical properties of MEV have been further supported by late-phase prosthesis expansion observed in a recent study by Kobari *et al*.^22^

Elevation of cardiac troponin level has been reported as a strong, independent predictor of 30-day mortality and a modest but significant predictor over 2 years of post-TAVI follow-up.^23^ We observed higher postprocedural troponin I levels in the MEV group than the SEV group, especially in the subgroups without PPI. In fact, the median troponin I levels even tended to be higher in the MEV group without PPI than in the SEV group with PPI (605±1423 vs 430±1034 ng/L in the MEV and SEV groups, respectively; p=0.065).

The increased cardiac damage caused by the MEV, including higher incidence of new PPI, NO-LBBB, late-phase prosthesis expansion and higher troponin I level after TAVI, raises the question regarding the impact of this long-term action of MEV on the LVOT in patients without PPI. Our results suggest that the survival of patients without PPI differs according to valve type. We assessed the relationship of new PPI and valve type using the Cox proportional hazards model. Despite the non-significant p value (0.089), we found a 1.76-fold higher mortality risk for patients with SEV and new PPI compared with patients with the same valve without PPI. In contrast, we observed a 0.73-fold lower mortality risk for patients with MEV and new PPI compared with patients with the same valve without PPI. One possible explanation for the higher cardiovascular mortality in the MEV group without PPI may be late-onset conduction disturbances related to prolonged pressure of the prosthesis on the conduction system passing through the LVOT. Indeed, a study by Urena *et al* suggested PPI as a protective factor against the occurrence of unexpected (sudden or unknown) death.^24^

### Limitations

This study had several limitations. First, the retrospective, observational, single-centre design has inherent limitations that should be considered before generalising the results. Despite the non-randomised nature of our study, the groups were well balanced (table 1). TAVI procedures (table 3) were accompanied by a low rate of complications, without significant variation between the groups. The lower number of balloon valvuloplasties in the MEV group was possible because of valve properties (radial force and capability for full retrieval). The higher percentage of open surgical access stems from the preferred strategy during this period of our TAVI programme, and it is most likely the cause of the lower haemoglobin levels after the procedure and longer hospital stays for patients with MEV (table 2). A significantly higher composite endpoint of device success was driven by the higher frequency of the AVGm gradient >20 mm Hg in the MEV group. This higher postprocedural gradient with MEV has been observed in other studies^6 25^ and is based on the intra-annular position of the prosthesis compared with the supra-annular placement of SEV. A significantly higher composite endpoint of early safety was driven by the higher frequency of new PPI associated with MEV. A high risk of conduction disturbances has also been associated with MEV.^8 9^ In our study, unlike MEV, AVGm>20 mm Hg and PPI were not identified as predictors of mortality.

Second, although we did not prove a difference in mortality between the MEV and SEV groups with implanted pacemakers, we cannot exclude the possibility of some variation. Nevertheless, our results indicated that the main mortality difference was observed in the groups without PPI.

Third, we did not find a difference of survival in the MEV group with and without PPI on the log-rank test; this would provide definitive proof of the association between PPI and survival.

## Conclusions

Despite several inherent limitations associated with the non-randomised design of this study, our results suggest that a higher cardiovascular mortality rate was associated with MEV over mid-term follow-up. On comparison of both groups according to the presence or absence of PPI, we observed a higher mortality risk in patients with MEV versus SEV without PPI. In contrast, we did not observe a mortality difference between the two groups when a permanent pacemaker was implanted.

## Data Availability

Data are available on reasonable request.
